# Adult-onset leukoencephalopathy, brain calcifications and cysts: a case report

**DOI:** 10.1186/1752-1947-7-151

**Published:** 2013-06-06

**Authors:** Yaming Wang, Gang Cheng, Chao Dong, Jianning Zhang, Yuhong Meng

**Affiliations:** 1 Department of Neurosurgery, PLA Navy General Hospital, Fuchenglu Road 6#, Haidian District, Beijing 100048, PR China; 2 Department of Pathology, PLA Navy General Hospital, Beijing, 100048, PR China

## Abstract

**Introduction:**

Leukoencephalopathy, brain calcifications and cysts, known as Labrune syndrome, is a rare syndrome. The etiology is unknown; in some cases it is difficult to differentiate from Coats plus syndrome and diagnosed as cerebroretinal microangiopathy with calcifications and cysts. We present the case of a patient with adult leukoencephalopathy, brain calcifications and cysts and discuss recently described entities in view of the relevant literature.

**Case presentation:**

A previously healthy 19-year-old Chinese man presented with weakness of his right limbs that rapidly worsened over a short interval. Computed tomography and magnetic resonance imaging showed numerous low-density cysts, calcifications, and abnormal signal change of white matter. A visual field examination showed irregular visual field defects in both eyes. A neuro-ophthalmologic examination did not find evidence of Coats retinopathy. A larger excisional biopsy was carried out and a diagnosis of leukoencephalopathy, brain calcifications and cysts was confirmed.

**Conclusions:**

We present an example of adult-onset leukoencephalopathy, brain calcifications and cysts and have expanded the clinical spectrum of features associated with this syndrome. Previous reports have not, to the best of our knowledge, previously reported visual field defects. Based on the latest findings, we believe that leukoencephalopathy, brain calcifications and cysts and Coats plus syndrome are genetically distinct entities.

## Introduction

Leukoencephalopathy, brain calcifications and cysts (LCC), also known as Labrune syndrome, is a rare syndrome characterized by extensive brain calcifications, leukodystrophy and the formation of parenchymal cysts. It was first described by Labrune *et al*. [1] in 1996. Clinical presentations are largely related to seizure or focal neurologic deficits consequent to progressive calcification or cystic expansion. We present the case of a patient with adult-onset LCC and discuss recently described entities in view of the relevant literature.

## Case presentation

A previously healthy 19-year-old Chinese man presented with weakness of his right limbs that rapidly worsened over a short interval. He had initially felt a weakness of his right lower limb six months prior to presentation. An X-ray of his right ankle at that time had been normal and no treatment was given. Two months ago, our patient felt a weakness of his right upper limb and the symptoms of his right lower limb worsened. At the same time, he developed fever (highest temperature, 39.5°C), blurred vision in his left eye and a mild episodic headache lasting for several minutes on each occasion. No nausea or vomiting occurred.

**Figure 1:**
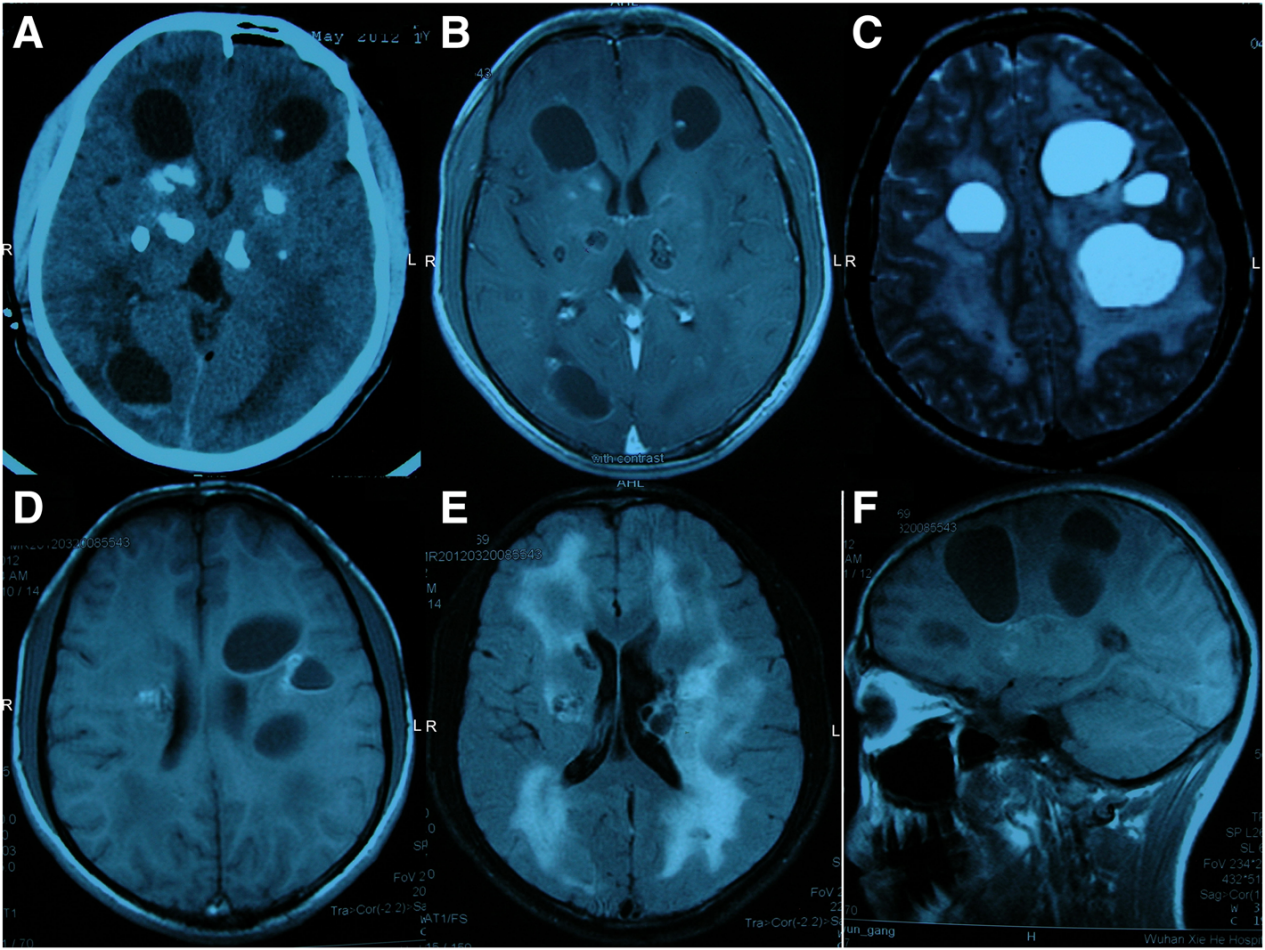
**Computed tomography and magnetic resonance imaging studies. (A) **Computed tomography showed numerous low-density cysts and calcifications scattered throughout both sides of the brain. **(B) **Axial T1-weighted post-gadolinium magnetic resonance imaging showed enhancement of the cyst wall. **(C) **Axial T2-weighted magnetic resonance imaging showed hyperintense cysts of different sizes. **(D) **Axial T1-weighted magnetic resonance imaging showed numerous cysts of various sizes. **(E) **Fluid-attenuated inversion recovery image showed hyperintensity in both sides of the brain. **(F)** SagittalT1-weighted magnetic resonance imaging showed numerous cysts of various sizes.

Computed tomography at an outside hospital showed numerous low-density cysts and calcifications scattered throughout both sides of his brain (Figure 1A). The boundary of each cyst was clear with a high-density ring, sometimes calcified. Brain magnetic resonance imaging demonstrated extensive cerebral white matter leukoencephalopathy. Numerous cysts of various sizes were scattered throughout his hemispheres, thalamus, basal ganglia and left ventricle (Figure 1B-F). The boundaries of the cysts were hyperintense on both T1- and T2-weighted images and gave a low signal on the fluid-attenuated inversion recovery image. Enhancement was observed in the lining of the cyst wall. The cystic content was heterointense in the T1-weighted and fluid-attenuated inversion recovery images.

**Figure 2:**
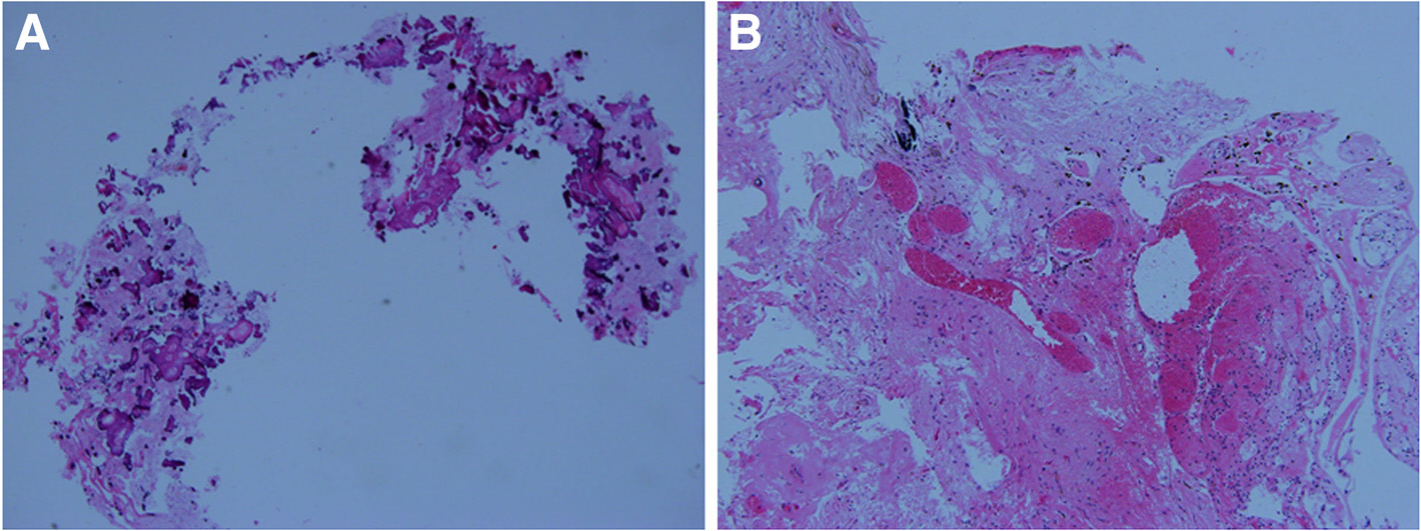
**Biopsy revealed a pronounced reactive gliosis with conspicuous formation of Rosenthal fibers and calcification, with frequent hemosiderin deposits. **(Hematoxylin and eosin ×100.) **(A) **Conspicuous formation of calcification. **(B) **Dilated capillaries with calcification, hemosiderin deposits and reactive gliosis.

**Figure 3:**
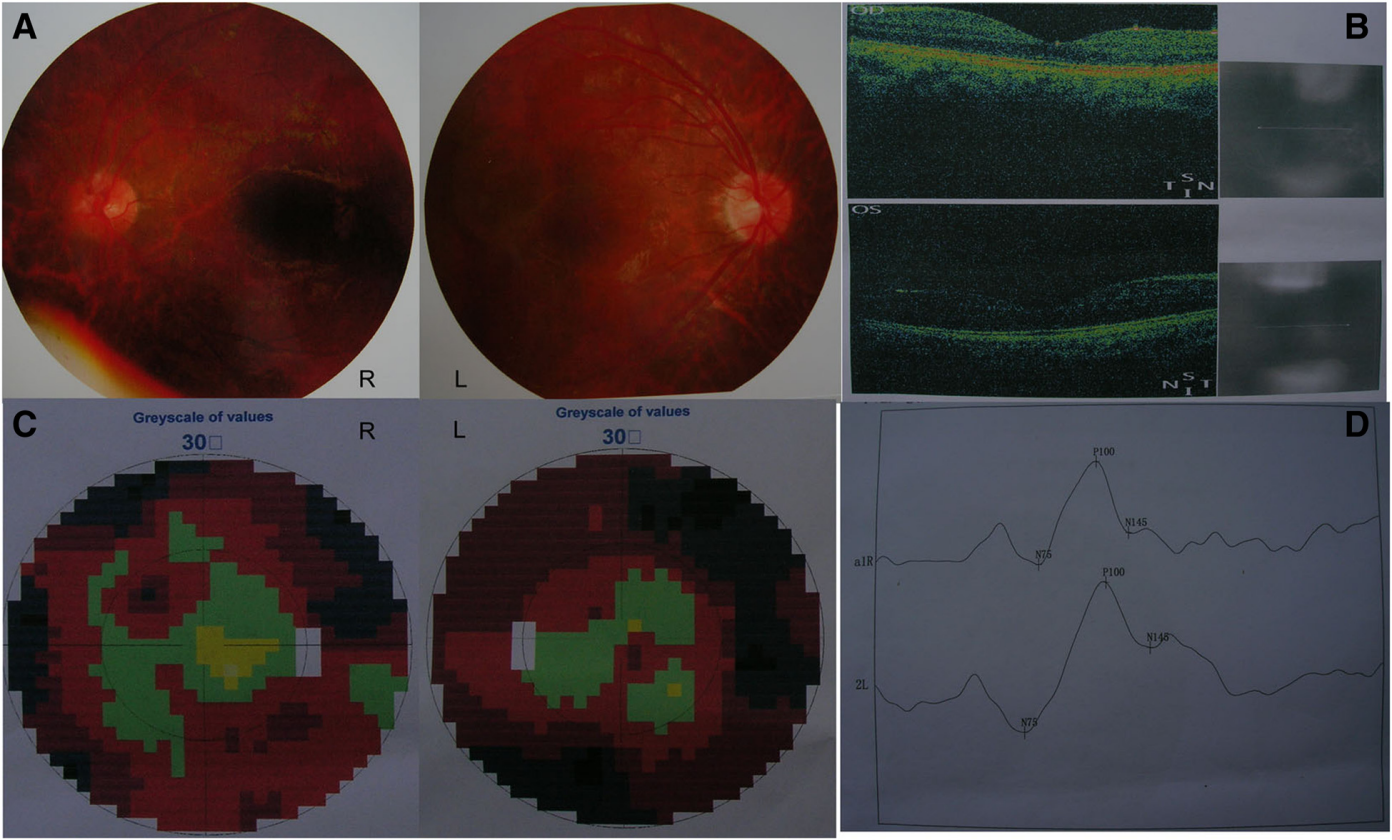
**Neuro-ophthalmologic examination, visual field examination and visual-evoked potential test of the patient. (A) **Retinal photograph showed a normal fundus. **(B) **Optical coherence tomography was normal. OD, right eye; OS, left eye. **(C) **A visual field examination showed an irregular visual field defect in both eyes. **(D) **Visual-evoked potentials disclosed a mild prolongation of P100 latency of the left eye and an increased amplitude of both eyes.

A physical examination showed that his vision was 0.15 in his left eye and 0.5 in his right eye. He had grade-4 strength in his right limbs and a positive Babinski sign on his right. The suspected diagnosis included parasitic infection, glioma and leukoencephalopathy. To make a correct diagnosis, we performed a large excisional biopsy of the left frontal cyst, including the adjacent brain tissue. Analysis of the cyst fluid did not suggest malignancy or infection (parasitic in particular). The biopsy specimen of his white matter and cyst wall revealed a pronounced reactive gliosis with conspicuous formation of Rosenthal fibers. In addition, focal hemosiderin deposits, which indicate previous hemorrhage, and microcalcifications were observed. Many ectatic vessels and angiomatous changes with cellulose-like degeneration were observed (Figure 2). A neuro-ophthalmologic examination was performed, but no evidence of Coats retinopathy was found (Figure 3A, B). A visual field examination showed irregular visual field defects of both eyes (Figure 3C). Visual-evoked potentials disclosed a mild prolongation of P100 latency of his left eye and an increased amplitude of both eyes (Figure 3D). The results of serological and immunological tests were within the normal range. We diagnosed our patient with LCC.

## Discussion

The clinical presentations of LCC include progressive extrapyramidal, cerebellar and pyramidal signs, slowed cognitive performances, and/or seizures. Depending on the anatomical location and distribution of these vessel and cyst changes, there can be widely varying cerebral, cerebellar and even spinal cord manifestations [2]. Clinical presentations of adult-onset cases are somewhat mild given the severe and progressive neurodegenerative process seen in the central nervous system on neuroimaging [3]. Because of the variety of clinical symptoms, it is difficult to differentiate LCC from Coats plus syndrome, another disorder that has similar manifestations. The most characteristic features of Coats plus syndrome are retinal telangiectasia and exudates; neuro-ophthalmologic examination is important in the differential diagnosis of LCC and Coats plus syndrome. In previous reports, Coats retinopathy was the only pathological change found during neuro-ophthalmologic examination, no visual field defects were mentioned. In our patient, irregular visual field defect of both eyes was the main clinical finding, but no evidence of Coats retinopathy was found. To the best of our knowledge, this is the first report of a visual field change in adult-onset LCC, and therefore expands the clinical spectrum of features of LCC.

Our patient showed a typical neuroimaging presentation of LCC- a triad of calcification in the deep cerebral nuclei and white matter, diffuse leukoencephalopathy, and multiple cystic brain lesions on brain imaging. Just like several other cases of adult-onset LCC, the disconnect between the highly worrisome magnetic resonance imaging findings and the relatively limited nature of clinical abnormalities was also found in our patient.

The etiology of LCC is largely unknown. There is overlapping extra-neurological manifestations between LCC and Coats plus syndrome, which makes it difficult to make a differential diagnosis. Coats plus syndrome was first described by Tolmie and co-workers in 1988 [4]. The most characteristic features of Coats plus syndrome are retinal telangiectasia and exudates (Coats disease), a distinctive pattern of intracranial calcification with associated leukodystrophy and brain cysts, and multi-system involvement [5-8] (Table 1). In 2004, Nagae-Poetscher *et al*. [5] described the cases of three children who had LCC, where one child also demonstrated Coats syndrome-like retinal changes, suggesting that LCC and Coats plus syndrome might be pathogenetically related (Table 1). During the next few years, several other authors reported other such overlapping cases. Linnankivi and co-workers [6] believe that Coats plus syndrome and LCC are manifestations of the same disease spectrum, termed cerebroretinal microangiopathy with calcifications and cysts. Recently, identification of biallelic mutations in the *CTC1* gene in the vast majority of patients with Coats plus syndrome but in none with LCC provides strong evidence that the two conditions are genetically distinct entities [9,10]. Anderson *et al*. [9] argued that the difference between LCC and Coasts plus is whether there are extra-neurological problems. Until now, there have been 19 reported cases of LCC and cerebroretinal microangiopathy with calcifications and cysts without extra-neurological problems, including this case [1-3,5-7,11-18] (Table 1).

**Table 1:** Summary of features of all reported patients with leukoencephalopathy, brain calcifications and cysts or with cerebroretinal microangiopathy with calcifications and cysts without extra-neurological problems

**Publication**	**Gender**	**IUGR**	**Age-of-onset (y)**	**Intracerebral calcification**	**Leukoencephalopathy**	**Cranial cysts**	**Cognitive decline/dementia**	**Seizure**	**Spasticity**	**Ataxia**	**Dystonia**
Nagae-Poetscher *et al*. (2004) [5]	F	+	0	+	+	+	-	+	+	+	+
Brenner *et al*. (2006) [15]	F	N/A	1 month	+	+	+	+	+	+	+	+
Labrune *et al*. (1996) [1]	F	-	3 months	+	+	+	+	+		+	+
Nagae-Poetscher *et al*. (2004) [5]	F	-	2				+		+	+	+
Briggs *et al*. (2008) [7]	M	-	8	+	+	+	-	-	+	+	+
Labrune *et al*. (1996) [1]	F	-	11	+	+	+	+	+	-	+	-
Liu *et al*. (2009) [16]	F	N/A	12	+	+	+	-	-	+	+	-
Linnankivi *et al*. (2006) [6]	M	N/A	14	+	+	-	+	+	+	+	-
Sener *et al*. (2006) [3]	M	-	15	+	+	+	-	+	+	-	-
Liu *et al*. (2009) [16]	M	-	19	+	+	+	-	-	-	-	-
Berry-Candelario *et al*. (2011) [11]	M	-	24	+	+	+	-	-	-	-	-
Daglioglu *et al*. (2009) [17]	M	N/A	26	+	+	+	-	-	-	+	-
Wargon *et al*. (2008) [18]	F	N/A	30	+	+	+	-	-	-	-	-
Kleinschmidt-Demasters *et al*. (2009) [2]	M	N/A	40	+	+	+	+	-	+	+	-
Corboy *et al*. (2006) [12]	F	N/A	44	+	+	+	+	+	-	+	-
Ummer *et al*. (2010) [14]	M	N/A	50	+	+	+	-	+	+	+	-
Kleinschmidt-Demasters *et al*. (2009) [2]	F	N/A	52	+	+	+	-	-	-	-	-
Kaffenberger *et al.* (2009) [13]	F	N/A	54	+	+	+	+	-	+	+	+

*IUGR *intrauterine growth retardation, *N/A *not available.

Because of the rarity of the disorder and the variety of clinical manifestations, a biopsy should be performed for tissue diagnosis. Based on the existing literature, the highest diagnostic yield likely involves draining the cyst and a biopsy of the cyst wall [11]. Histopathology of LCC is characterized by angiopathy, calcification and Rosenthal fibers [1], and the tissue from our patient showed all these features. Based on histopathologic findings, some authors believe that cerebral microangiopathy is the possible etiology of LCC [6,12]. Kaffenberger *et al*. suggested that distinct but overlapping pathophysiological mechanisms (demyelination and edema) lead to leukoencephalopathy, calcifications and cysts [13]. Berry-Candelario *et al*. also suggested that these abnormalities may constitute an umbrella term that encapsulates distinct disease entities, including microangiopathy, aberrant myelination, or multiple central nervous system injuries or insults in the context of congenital abnormalities [11]. Armstrong *et al*. found both growth and shrinkage in cysts over time in their neuroimaging of a patient. They believed that the decrease in size of some cysts over time may suggest a more complex pathological finding than has been previously theorized [19].

## Conclusions

This is an example of adult-onset LCC and expands the clinical spectrum of features of LCC. Based on the latest findings, we believe that LCC and Coats plus syndrome are genetically distinct entities.

## Consent

Written informed consent was obtained from the patient for publication of this case report and accompanying images. A copy of the written consent is available for review by the Editor-in-Chief of this journal.

## Abbreviations

LCC: leukoencephalopathy, brain calcifications and cysts.

## Competing interests

The authors declare that they have no competing interest.

## Authors’ contributions

YW analyzed and interpreted the patient data. GC was a major contributor in writing the manuscript. CD obtained and analyzed the patient data. JZ was a major contributor in interpreting the data and manuscript revision. YM performed the histological examination. All authors read and approved the final manuscript.
